# Assessment of the Influence of Additives on the Mechanical Properties and Machinability of Al-11%Si Cast Alloys: Application of DOE and ANOVA Methods

**DOI:** 10.3390/ma15093297

**Published:** 2022-05-04

**Authors:** Yasser Zedan, Victor Songmene, Agnes M. Samuel, Fawzy H. Samuel, Herbert W. Doty

**Affiliations:** 1 Département de Génie Mécanique, École de Technologie Supérieure, Montréal, QC H3C 1K3, Canada; victor.songmene@etsmtl.ca; 2 Département des Sciences Appliquées, Université du Québec à Chicoutimi, Chicoutimi, QC G7H 2B1, Canada; agnesmsamuel@gmail.com (A.M.S.); fawzy-hosny.samuel@etsmtl.ca (F.H.S.); 3 General Motors Global Technology Center, Warren, MI 48093-2350, USA; herb.doty@gm.com

**Keywords:** Al-Si alloys, alloying elements, mechanical properties, machinability, DOE, ANOVA

## Abstract

In the present study, the statistical design of experiments (DOE) method was applied to study and control the properties of near-eutectic Al-11%Si alloys. In this study, we developed regression equations between response variables, including hardness, yield stress, ultimate tensile stress, elongation, total cutting force, cutting power, and tool life, and varying factors which included the percentage of the alloying element in the composition and the modification level. These equations may be analyzed quantitatively to acquire an understating of the effects of the main variables and their interactions on the mechanical behavior and the machinability of the alloy under investigation. Analysis of variance (ANOVA) was performed to verify the fit and adequacy of the developed mathematical models. The results show that increasing the levels of Cu and Fe results in an increase in hardness, yield stress and ultimate tensile strength in both modified and non-modified alloys. On the other hand, both Cu and Fe appear to affect the elongation adversely, whereas the Sr level shows a positive effect on the elongation percentage. We found that the Sr level had the most significant effect on the cutting forces and cutting power, followed by Fe and Cu contents.

## 1. Introduction

Aluminum-silicon alloys are normally employed for the fabrication of automotive components due to their excellent mechanical behavior. Their relatively low density, compared to those made of steel or cast iron, has made them more convenient for use in transmission cases and intake manifolds, as well as engine block parts [1]. In turn, the enhanced quality of aluminum alloy workpieces requires intensive investigations in terms of their microstructure, as well as from the point of view of ambient- and high-temperature performance [2].

The main micro-constituents that have been observed in this microstructure are eutectic Si particles. The size and distribution of these particles depend on two main parameters: chemical treatment by Sr and the application of high solidification rates [3]. In addition, other phases that would be observed are Fe-based intermetallics (with β-platelet or α-Chinese script morphologies), together with Mg_2_Si and Al_2_Cu, and other complex phases [4,5]. Zedan et al. [6] and Pathak et al. [7] examined the influence of Fe-based intermetallics on the machinability of Al-Si alloys with different levels of Fe and Mn. The results revealed that Fe phases, in particular sludge, would cause significant deterioration of the cutting tools used, coupled with a marked increase in the machining power required and hence in machining costs.

When Cu and Mg constitute a significant part of the employed aluminum alloys, their interaction with other elements in the alloy could result in improved properties. In addition, wearing of the cutting tool is not an issue except when their volume fraction is as high as 50%, particularly in regard to the formation of insoluble Al–Cu–Mg phases [8,9,10,11]. The application of the statistical design of experiments (DOE) method enabled the designers to understand the role of the factors that would determine the design of the final product. The use of the DOE method has been the subject of many studies, leading to marked achievements in the area of the development of computer science [12].

Several researchers [13,14,15,16,17] have investigated the behavior of Al-based alloys using the DOE method. These researchers reported that tool life is primarily influenced by the materials and strength of the workpiece. A model was designed by Othman et al. [18] and Khorasani et al. [19], which indicated that the thrust force and torque required for drilling the last hole is approximately 50% higher than those required for drilling the first hole.

In the present study, an attempt was made to investigate the effect of compositional variations, including modification of the Cu content, Fe content and Sr content, on the mechanical properties and machinability of heat-treated Al-11%Si near-eutectic alloy. The responses measured in the experiments were hardness, yield stress, ultimate tensile strength, percentage elongation, total drilling forces, drilling power, and drill life as a function of the number of the drilled holes up to the point of drill fracture. A three-factor, two-level full factorial design was adopted for analyzing the results. A procedure was developed to establish the relationship among the investigated parameters by incorporating (i) a standardized Pareto chart; (ii) main and interaction graphs; and (iii) the analysis of various variables (ANOVA) method.

## 2. Scheme of Investigation

Several factors could influence the mechanical properties of Al-Si alloys and their machinability performance, such as (i) the percentage composition of the alloying element, (ii) the heat treatment, (iii) the melt treatment, and (iv) the casting mode. Prior to the present investigation, the role of alloying elements in determining the final microstructure and hence the mechanical properties of alloys was examined. The main elements studied were Fe, Mn, Cu, and Mg. Consequently, alloys with potential applications were considered for the following purposes:

1. Identifying the important factors which influence the characteristics of Al–Si casting alloys;
2. Finding the upper and lower limits of the factors identified;
3. Developing the experimental design matrix using the design of experiments method;
4. Conducting the experiments as per the design matrix;
5. Developing regression equations between the response variable and the independent factor;
6. Assessing the factors and their effects using a standardized Pareto chart; and
7. Analyzing the results using analysis of variance (ANOVA).

### 2.1. Developing the Experimental Design Matrix

For this study, three parameters were varied for two levels: Cu (2.25% and 3.5%), Fe (0.5% and 1%), and Sr-level (0 and 200 ppm). To carry out the experiments, the statistical design of experiments method was used. This significantly reduces the number of experiments and the time required, compared to experiments assessing one factor at a time. These designs are labeled 2^n,^, where n is the number of factors that may be evaluated in the full factorial design, i.e., 2^3^ = 8 trials in the experiment. Table 1 represents the notations, units, and levels of factors which were varied during the present study.

### 2.2. Evaluation of Response Variables 

The measured responses in these experiments were the mechanical properties and machining behavior of investigated alloys. The mechanical properties evaluated were hardness, yield stress (YS), ultimate tensile strength (UTS), and percentage elongation (%El), whereas the machining response variables were the total drilling force, drilling power, and drill life (which was defined by the number of holes drilled up to the point of drill fracture). Table 2 presents the parameters analyzed, along with their codes. The mechanical properties were evaluated using hardness and tensile tests, as provided in detail in Section 3, in which we present the experimental procedures.

On the other hand, the machinability response variable used in this work was evaluated as follows:

1. Total Cutting Force and Power

A Kistler model 9255B, 6-component piezoelectric quartz crystal dynamometer was used during drilling tests for the online measurement of the cutting forces and moments. The total cutting force and moment were calculated using the Matlab signal processing program [20]. The signals obtained were processed in such a way that the mean components of the cutting force (Fx_m_, Fy_m_, and Fz_m_) and moment (Mx_m_, My_m_, and Mz_m_), as well as their corresponding standard deviations (σ_Fxm_, σ_Fym_, σ_Fzm_, σ_Mxm,_ σ_Mym_, and σ_Mzm_), were calculated for each hole using the following set of equations:

Ft_m_ = [(Fx_m_)^2^ + (Fy_m_)^2^ + (Fz_m_)^2^]^1/2^ Mt_m_ = [(Mx_m_)^2^ + (My_m_)^2^ + (Mz_m_)^2^]^1/2^ (1)

The standard deviations σ_Ftm_, σ_Ftm_ of the total mean cutting force and moment, respectively, were calculated as follows [1]:

σ_Ftm_ = [(Fx_m_)^2^ (σ_Fxm_)^2^ + (Fy_m_)^2^ (σ_Fym_)^2^ + (Fz_m_)^2^ (σ_Fzm_)^2^]^1/2^/Ft_m_ σ_Mtm_ = [(Mx_m_)^2^ (σ_Mxm_)^2^ + (My_m_)^2^ (σ_Mym_)^2^ + (Mz_m_)^2^ (σ_Mzm_)^2^)]^1/2^/Mt_m_ (2)

Eventually, the drilling force and moment and their standard deviations were found for each test block as the mean values calculated over the respective values of 180 holes drilled in the same block. Consequently, the drilling power was calculated in this study by employing the following expression:

Pc = (π*Mz_m_*n)/30 (3)

where n represents drill speed.

2. Tool Life Criteria

In this study, each alloy condition was tested with a new drill until it broke. It should be mentioned here that each drilling test was carried out at least two or three times to validate the results regarding drill life. A drill life of 2500 holes, i.e., 14 test blocks, was targeted for each alloy condition.

Based on the previous machinability studies carried out by our group, the drilling tests were carried out as follows: if the drill broke down during the drilling process, one of two options was followed: (i) drilling was halted and then the test was changed to another alloy condition, or (ii) in the case that the drill broke as a result of the presence of a defect or large inclusion, the test was resumed for the remaining blocks of the same alloy condition using a new drill. All alloy conditions were tested under the same drilling conditions [20].

## 3. Experimental Procedures

All experiments were conducted on experimental Al-11%Si alloy, which was received in the form of 12.5 kg ingots. The chemical composition of the base alloy was 10.8% Si, 2.24% Cu, 0.31% Mg, 0.46% Fe, 0.49% Mn, 0.014% Sr, and 0.057% Ti, with Al as a balance. Melting was carried out in an SiC crucible with a 120 kg capacity, using an electrical resistance furnace in which the melting temperature was maintained at 750 °C ± 5 °C. At this temperature, measured amounts of Cu, Fe, and Sr were added. All melts were degassed using pure dry argon injected into the melt for ~15 min by means of a rotating graphite degassing impeller (125 rpm), to ensure homogenous mixing of the additions.

For each set of melt conditions, identical castings were prepared for tensile and machining testing. The melt was poured at ~735 °C into the following molds, which had been preheated to 450 °C:

(i) An ASTM B-108 permanent mold (five bars per each condition);
(ii) A waffle-plate graphite-coated metallic mold to obtain castings for machinability test blocks (eighteen machinability test blocks per each condition).

The tensile and machinability specimens were solution heat-treated at 495 °C for 8 h, then quenched in warm water at 65 °C, followed by artificial treatment at 180 °C for 5 h (i.e., the bars were T6 tempered). Both the solution and aging heat treatments were carried out in a forced-air Blue M electric furnace, equipped with a programmable temperature controller accurate to within ±2 °C.

Hardness measurements were carried out on the heat-treated samples using a Brinell hardness tester, with a steel ball of 10 mm in diameter and a load of 500 kg applied for 30 s. Four blocks were randomly selected from among the eighteen test blocks prepared for each alloy condition. The average hardness value for the four blocks selected per alloy was then obtained and designated as representing the hardness value for that alloy condition.

Tensile testing was conducted at room temperature using an MTS servo-hydraulic universal testing machine. The average yield strength (YS), ultimate tensile strength (UTS), and elongation to fracture (%El) values obtained from the five samples tested were considered to be the values representing a specified alloy/condition. It should be kept in mind that all of these alloys were mechanically tested in order to acquire an understanding of the effects of the additives on the mechanical properties at the same specific T6 heat-treated conditions which were applied to the machinability test blocks.

Drilling tests were performed using a Makino A88E high-speed horizontal machining center with maximum power of 40 HP (30 kW) and a maximum rotation speed of 18,000 rpm under fixed machining conditions in terms of speed, feed, length of cut, tool type, and coolant, as applied to the examination of the alloys under discussion. The drilling tests were carried out at rotational speeds of 11,000 rpm using a feed rate of 1.117 m/min, with each hole being 28.38 mm deep, as provided in Table 3. A synthetic metalworking fluid concentrate composed of 5% cutting fluid + 95% liquid, known as CIMTECH^®^ 310, was pumped at high pressure through the drill to ensure adequate cooling and chip evacuation.

## 4. Assessing the Factors and Their Effects

Assessing the factors and their effects on the mechanical properties and machining performance of experimental Al-11%Si alloys was carried out through the use of (i) a mathematical model; (ii) a standardized Pareto chart; and (iii) the analysis of variance (ANOVA) technique.

### 4.1. Mathematical Modeling

The purpose of developing the mathematical model relating the response variables (mechanical properties and machining behavior) and the metallurgical factors (percentage composition of the alloying element and modification level) was to conduct a quantitative analysis to acquire an understanding of the effects of the variables and their interactions on the properties of Al–Si casting alloys. According to the two-level experimental design, a non-linear object may be approximated by means of a nonlinear regression function, as shown in Equation (4). The aim of the analysis was to find out the effect of independent variables on the response

Y = b_0_ + b_1_ X_1_+ b_2_X_2_ + b_3_X_3_ + b_1_b_2_X_1_X_2_ + b_1_b_3_X_1_X_3_ + b_2_b_3_ X_2_X_3_ (4)

where Y is the response variable (hardness, yield stress, ultimate tensile strength, percentage elongation, total drilling force, drilling power, tool life); b_0_, b_1_, b_2_, and b_3_ are constants representing the effects of the main variable; and b_1_b_2_, b_1_b_3_, and b_2_b_3_ represent the respective interaction factor. X_1_, X_2_, and X_3_ are coded values of the factors of copper (Cu), iron (Fe), and strontium (Sr) content, respectively. For the convenience of recording and processing the experimental data, the upper and lower levels of factors or parameters are coded as +1 and −1. The coded value of any intermediate level can be calculated by using the following expression:

(5) $$X_{i\;}=\frac{X-\left[\frac{X_{\max+X_{\min}}}{2}\right]}{\left[\frac{X_{\max-X_{\min}}}{2}\right]}$$

where *X*_max_ is the upper level of the parameter, *X*_min_ is the lower level of the parameter, and *X_i_* is the required coded values of the parameter of any value of *X* from *X*_min_ to *X*_max_.

In the present study, STATGRAPHICS Centurion XVI software was employed to calculate the principal parameters and the interactions between the independent variables and the response variables using an experimental Al-11%Si alloy. In this case, the level of variables changed from level −1 to level +1. In addition, coded values were inserted into Equation (4) in order to compute the levels of response variables. The equations are non-linear, along with multiple binary and ternary coefficients. Both experimental factors and the response variables are listed in Table 4.

The standard deviation associated with the average value of the response variables is also reported. By processing the data provided in Table 4, regression Equations (6)–(12) were developed for the hardness, YS, UTS, %El, Ft_m_, Pc, tool life, and the variation of a number of different factors as follows:

Y_1_ (BHN) = 114.75 + 1.75X_1_ + 0.25X_2_ + 0.5X_3_ − 0.25X_1_X_2_ + 4X_1_X_3_ + X_2_X_3_ (R^2^ = 90.99%) (6)

Y2 (Y.S) = 350 + 10X_1_ + 5.25X_2_ + 7.75X_3_ + 17.25X_1_X_2_ + 5.75X_1_X_3_ − 4X_2_X_3_ (R^2^ = 80.68%) (7)

Y_3_ (UTS) = 383.5 + 11X_1_ + 2X_2_ − 4X_3_ + 17.5X_1_X_2_ −5.5X_1_X_3_ − 4.5X_2_X_3_ (R^2^ = 94.199%) (8)

Y_4_ (El) = 0.6975 − 0.0675X_1_ − 0.0525X_2_ + 0.105X_3_ + 0.0375X_1_X_2_ − 0.06X_1_X_3_ − 0.03X_2_X_3_ (R^2^ = 94.79%) (9)

Y5 (Ft_m_) = 480 + 18.5X_1_ + 22X_2_ + 48.5X_3_ + 16.5X_1_X_2_ + 3X_1_X_3_ + 11.5X_2_X_3_ (R^2^ = 93.522%) (10)

Y6 (Pc) = 2.187 − 0.325X_1_ − 0.285X_2_ + 0.51X_3_ − 0.2775X_1_X_2_ + 0.103X_1_X_3_ + 0187X_2_X_3_ (R^2^ = 91.528%) (11)

Y7 (Tool life) = 1152 + 93X_1_ + 86.25X_2_ + 92.25X_3_ + 341.25X_1_X_2_ − 414.25 X_1_X_3_ − 339.5X_2_X_3_ (R^2^ = 89.81%) (12)

where the values of X_1_, X_2_, and X_3_ can be decoded using the following relations:

X_1_ = (%Cu − 2.75)/0.75, which ranged between 2.25% and 3.5%;

X_2_ = (%Fe − 0.75)/0.25, which ranged between 0.5% and 1%;

X_3_ = (Sr − 100)/100, which ranged between 0 and 200 ppm.

In general, the influence of the addition of copper (Cu) on the response variables Y_H_,Y_YS_, Y_UTs_, Y_%El_, Y_Ftm_, Y_Pc_, and Y_tool life_ is represented by the coefficients b_1_, b_1_b_2_, and b_1_b_3_. A comparison of the values of these coefficients indicates that (i) the coefficient b_1_ is of crucial importance; (ii) when considering the effect of the addition of (Fe) on the response variables, the coefficients b_2_, b_1_b_2_, and b_2_b_3_ should be taken into account; in this particular case, the coefficient, b_2_, is of major significance; and (iii) the addition of strontium (Sr) has an effect on the response variables as a result of the coefficients b_3_, b_1_b_3_, b_3_b_2_; b_3_ appears to have a greater influence on the values of the response variables.

The regression equations which were created for this study show varying degrees of accuracy. Correlation coefficients (R^2^, $R_{Adj}^{2}$) are given in Table 5. For example, the value of R^2^ = 0.9099 for hardness indicates that 90.9% of the total variations are explained by model and 9.1% is accounted for either by variables which are assumed to be constant or by the inability of the data to be modeled by a quadratic equation. The adjusted R^2^ value is a statistic that is adjusted for the “size” of the model, that is, the number of factors (terms). The value of $R_{Adj}^{2}\;$= 0.85944 indicates that 85.94% of the total variability is explained by the model after considering the significant factors. As shown in Table 5, the models for hardness, UTS, %El, Ft_m_, and Pc had high multiple correlation coefficients, whereas those for YS and tool life were slightly low, suggesting that these two variables were sensitive to some factors which were not included within the scope of this study.

The validity of the equations was checked by performing random experiments in the range of the variation of Cu, Fe, and Sr contents. Table 6 and Table 7 provide a comparison between the calculated values of the mechanical and machining properties obtained from Equations (6)–(12) and the values obtained experimentally from the random runs. An examination of the results indicates that there was a close match between the properties obtained by performing random experiments and those calculated using the respective regression equations. The preceding operation was carried out by inserting the reduced values of the parameters corresponding to the random experiments into the respective equations. The closeness of the match indicates that the equations were sufficiently accurate within an acceptable range of variations in the variables.

From the proposed two-level factorial design experiments on the properties of near-eutectic Al-11%Si alloys, it is obvious that by using this type of polynomial regression equation, the effect of each of the individual variables and those of their interactions on the mechanical and machining properties may be obtained. Modifications may suitably be applied to the equation model in order to clarify the responses of the properties of the samples beyond the specified range. Finally, for a better understanding of the effects of individual variables and their interaction on the mechanical and machining properties, a higher level of factorial experimental design was suggested, wherein the influence of other parameters, such as heat treatment, casting mode, cooling rate, and so forth, could be examined.

### 4.2. Standardized Pareto Chart

**Figure 1:**
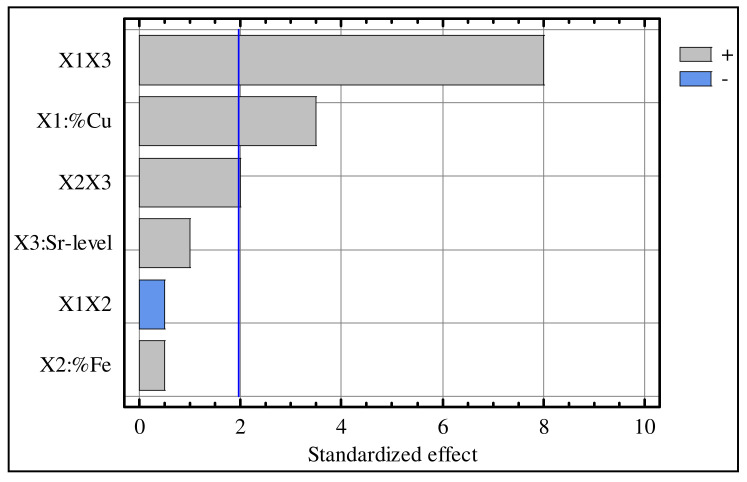
Pareto charts of the standardized effects for hardness data.

A standardized Pareto chart is a horizontal bar chart plotting values in descending order. The length of each bar is proportional to the values of the estimated effect. Figure 1 shows the Pareto chart of the standardized effects for the hardness data with a confidence level of 95%, in which the most significant effects corresponded to the interaction effect between the Cu content and Sr level (X_1_X_3_). Next in significance were the Cu-content (X_1_) and the interaction effect between the Fe content and Sr level (X_2_X_3_). However, the effects of the coefficients X_3_, X_1_X_2_, and X_2_ were found to be insignificant.

**Figure 2:**
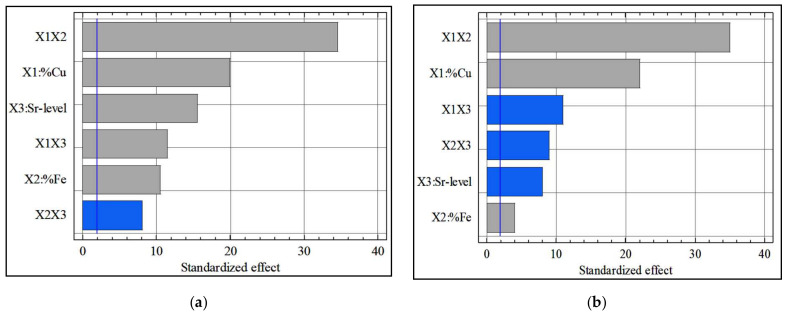
Pareto charts of the standardized effects for (**a**) yield stress data and (**b**) ultimate tensile strength data.

Figure 2a,b show the effect of alloying elements on the yield stress (YS) and ultimate tensile strength (UTS) values. It can be noted that, of the three alloying elements, Cu had the greatest effect by increasing the YS and UTS values. Cu is thus a superior strengthener and its addition as an alloying element is desirable, whereas the Sr level and Fe content also increased the strength but this effect was mild.

**Figure 3:**
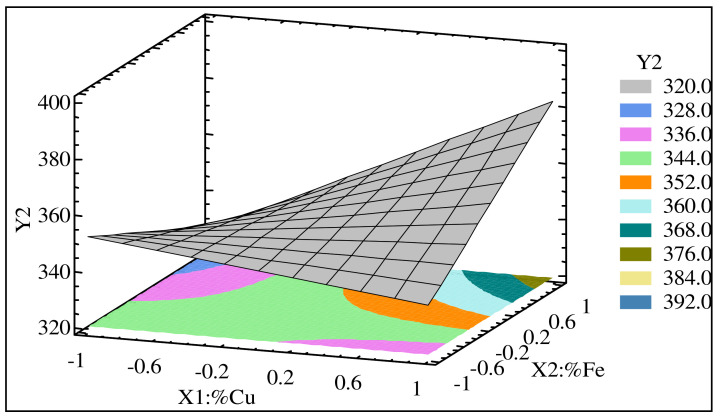
Regression model for yield stress (YS) as a function of Cu and Fe content in heat-treated Al-11%Si alloys containing 100 ppm Sr.

It should be noted that the presence of a number of binary interactions indicates the formation of various intermetallic compounds. Therefore, several interaction effects were present, and they may have had a significant effect on the strength value. These interaction effects included the interaction between Cu and Fe, namely, X_1_X_2_, and the interaction between Cu and the Sr level, namely, X_1_X_3_. Figure 3 shows a three-dimensional (3D) representation of the response surface of YS as function of the coded values of Cu content (X_1_) and Fe content (X_2_) for alloys containing medium level of Sr (100 ppm). These results point to the maximum values for YS occurring at high levels of Cu (+1) and Fe (+1), whereas the minimum values of YS occurred at low levels of Cu and Fe for alloys containing 100 ppm of Sr. The average values of yield stress were calculated for all combinations. Using these values, interaction graphs were drawn for each combination.

**Figure 4:**
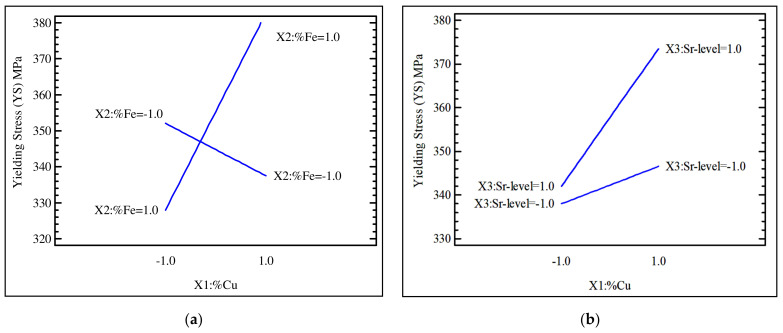
(**a**) Interactive effect of %Cu (X_1_) and %Fe (X_2_); (**b**) interactive effect of %Cu (X_1_) and Sr-level (X_3_) on the yield stress values (YS in MPa).

Figure 4a shows the interaction coefficient (X_1_X_2_) between Cu content and Fe content, in which the yield stress was found to increase significantly with an increase in the Cu content from a low level (−1) to a high level (+1) at a high level of Fe (+1), i.e., alloys containing 1% Fe. On the other hand, the yield stress decreased slightly with an increase in the Cu content at a low level of Fe, i.e., alloys containing 0.5% Fe. Figure 4b represents the interaction coefficient (X_1_X_3_) between the Cu content and the Sr level; the yield stress was found to increase significantly with an increased level of Cu content from 2.25% to 3.5% in the modified alloy which contained a high level of Sr. On the other hand, the yield stress increased slightly with an increase in the Cu content in the non-modified alloy. It was also observed that the modified alloys exhibited yield stress values higher than those of non-modified alloys within all levels of Cu contents, as shown in Figure 4b.

**Figure 5:**
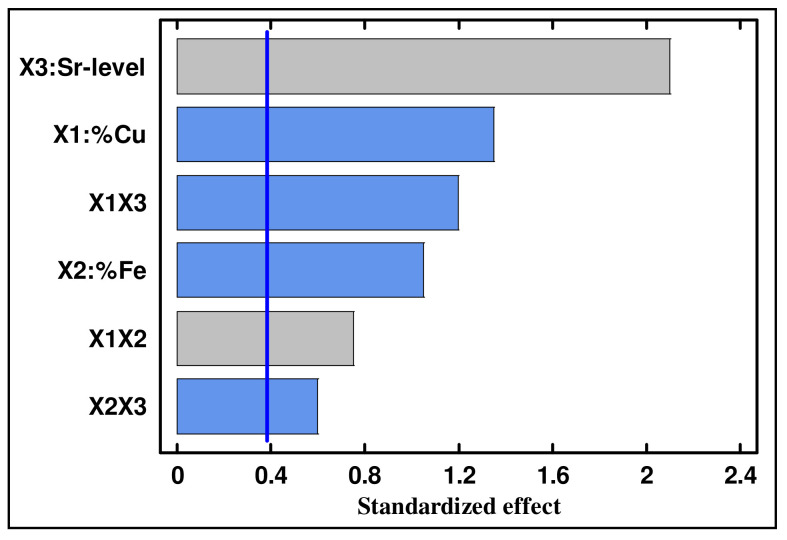
Pareto charts of the standardized effects for the elongation percentage.

Figure 5 shows that the elongation percentage (%El) was highly sensitive to alloy composition. In this Figure, it can be observed that most of the variables contributed negatively to the elongation percentage. Both Cu and Fe appeared to affect the elongation adversely, whereas the Sr level (X3) showed a positive effect on the elongation percentage.

**Figure 6:**
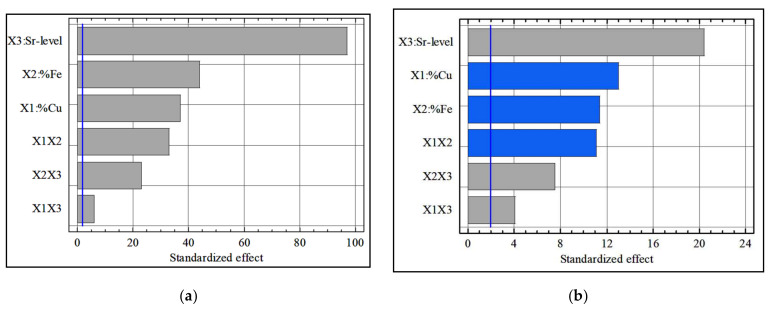
Pareto charts of the standardized effects for (**a**) total cutting force data and (**b**) cutting power data.

**Figure 7:**
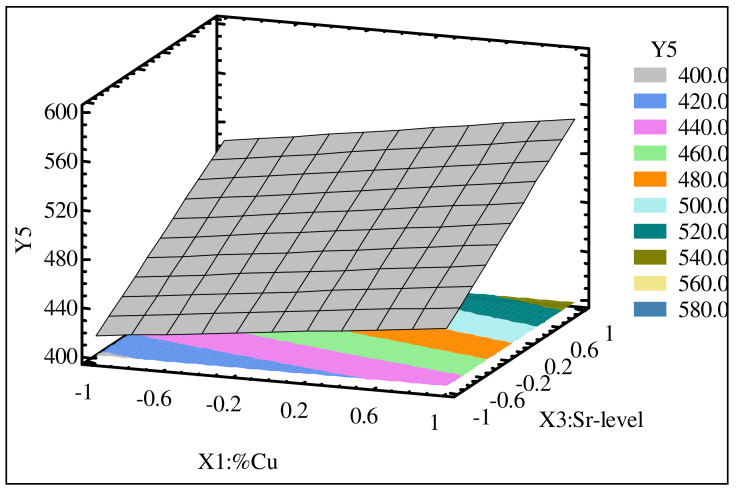
Regression model for total cutting force (F_tm_) as a function of Cu and Sr content of heat-treated Al-11%Si alloy containing 0.5% Fe.

Figure 6a,b show the relative significance of the independent parameters on the cutting forces and cutting power (Pc). The Sr level (X_1_) had the most significant effect, followed by the Fe content (X_2_) and Cu content (X_3_). Next in significance were the interactions between these parameters. Figure 7 shows the regression model for cutting force as a function of the Cu content (X_1_) and Sr level (X_3_) for alloys containing 0.5% Fe. This figure once again points to the fact that the maximum values for the cutting force prevailed with Sr and Cu contents greater than 0 ppm and 2.5%, respectively. On the other hand, the minimum cutting force occurred at low levels of Sr and Cu.

**Figure 8:**
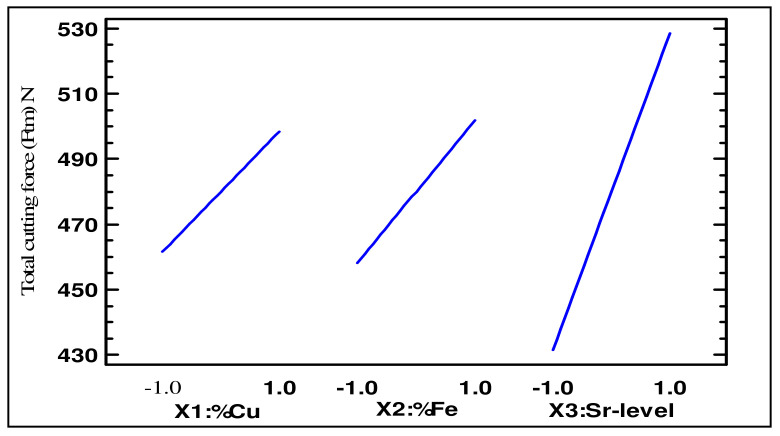
Main effects plots for total cutting force (F_tm_ in N) as a function of Cu, Fe, and Sr content for heat-treated Al-11%Si alloys.

As also observed from the main effects plot of total cutting force as function of Cu, Fe, and Sr contents, the cutting force was found to increase significantly with an increase in the level of Sr for a constant level of Cu and Fe. The cutting force was also found to increase with an increase in the level of Cu and Fe and a constant level of Sr, as clearly shown in Figure 8.

**Figure 9:**
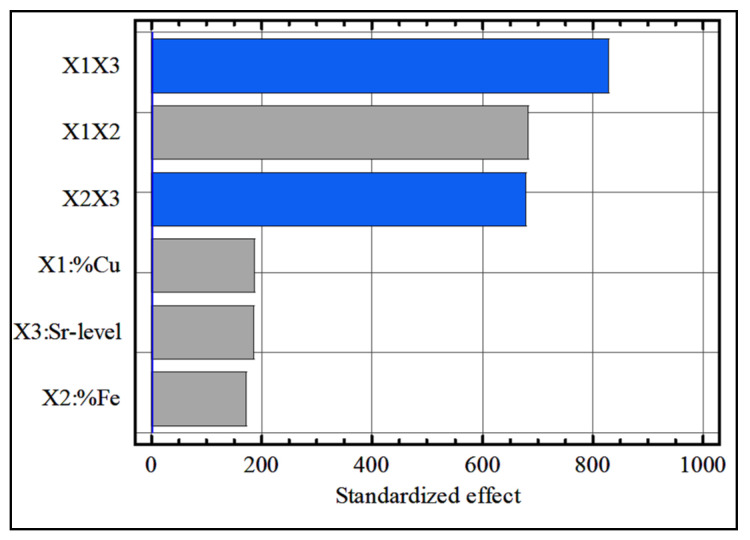
Pareto chart of the standardized effects for tool life data.

The same procedures were applied on tool life, resulting in a Pareto charts with a confidence level of 95%, as shown in Figure 9. It was also observed that the binary interactions between X_1_, X_2_, and X_3_ had significant effects on the tool life. As shown in this figure, the interaction coefficients of X_1_X_3_, X_1_X_2_ and X_2_X_3_ were the most significant in comparison to independent variables X_1_, X_2_, and X_3_. This fact may be attributed to the formation of complex insoluble phases between Cu, Fe, Si, and Al.

### 4.3. Analysis of Variance (ANOVA) Technique

An ANOVA summary table is commonly used to summarize the testing of a regression model, testing of significant factors and their interaction, and lack-of-fit testing. If the *p*-values in the ANOVA table are less than 0.05, then the factors (and the interaction of factors) are said to be significant. Finally, the % F-value column is used in the ANOVA summary table and this often serves as a rough but effective indicator of the relative importance of each model term.

The results of the ANOVA for yield stress are shown in Table 8; this analysis was carried out for a level of significance of 5%, i.e., for a confidence level of 95%. As shown in Table 8, Table 9 and Table 10, the interaction coefficients (X_1_X_2_), the Cu content (X_1_), and the Sr-level (X_3_) contributed 34.5%, 20%, and 15.5% to the total variability of the model, respectively. These results show that the alloying elements interacted with each other to a significant degree. Similarly, the results of the analysis of variance (ANOVA) for total cutting force and tool life are shown in Table 7 and Table 8, respectively.

## 5. Discussion

The properties of alloys may be improved by adding Cu, Fe, and Sr to the alloys. Mohamed et al. [21,22] reported on the changes in the microstructure (i.e., with respect to intermetallics and silicon particle characteristics) with the addition of Fe, Mn, Cu, Sr, and Mg to Al-10.8%Si near-eutectic alloys. The results showed that increasing the level of Mg and Cu in the Sr-containing alloys produced larger Si particle sizes, thus, in effect, diminishing the modifying influence of Sr. Among intermetallics, Al_2_Cu phase particles were more or less completely dissolved in the Al matrix after solution heat-treatment, whereas the β-Fe phase underwent partial dissolution and Al_2_Cu_2_Mg_8_Si_6_, α-Fe intermetallic phases and sludge phases persisted after 8 h of solution time in all the samples. 

**Figure 10:**
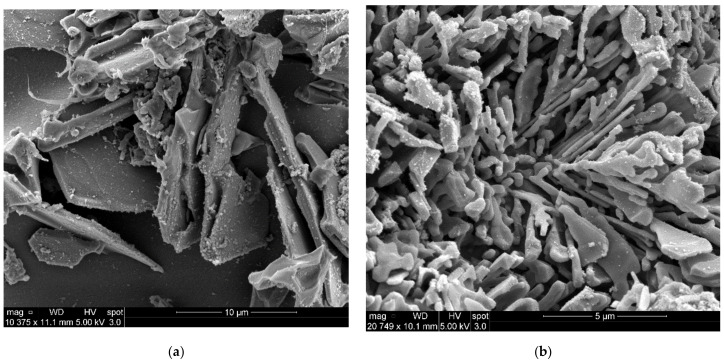
Backscattered electron micrographs showing the effects of the addition of Sr on Si morphology in grain-refined Al-11%Si alloy in the as-cast condition. (**a**) Alloy without Sr (non-modified alloy); (**b**) Alloy with 200 ppm Sr (high Sr content).

**Figure 11:**
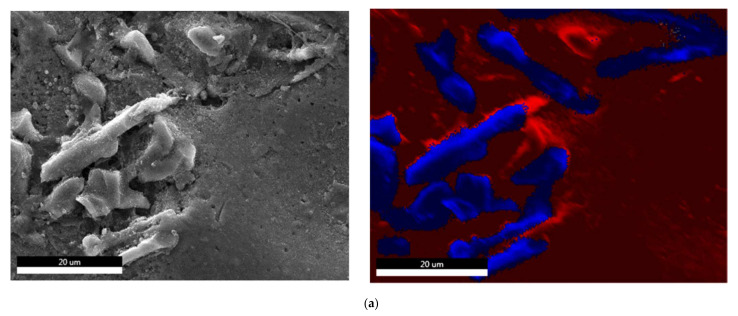

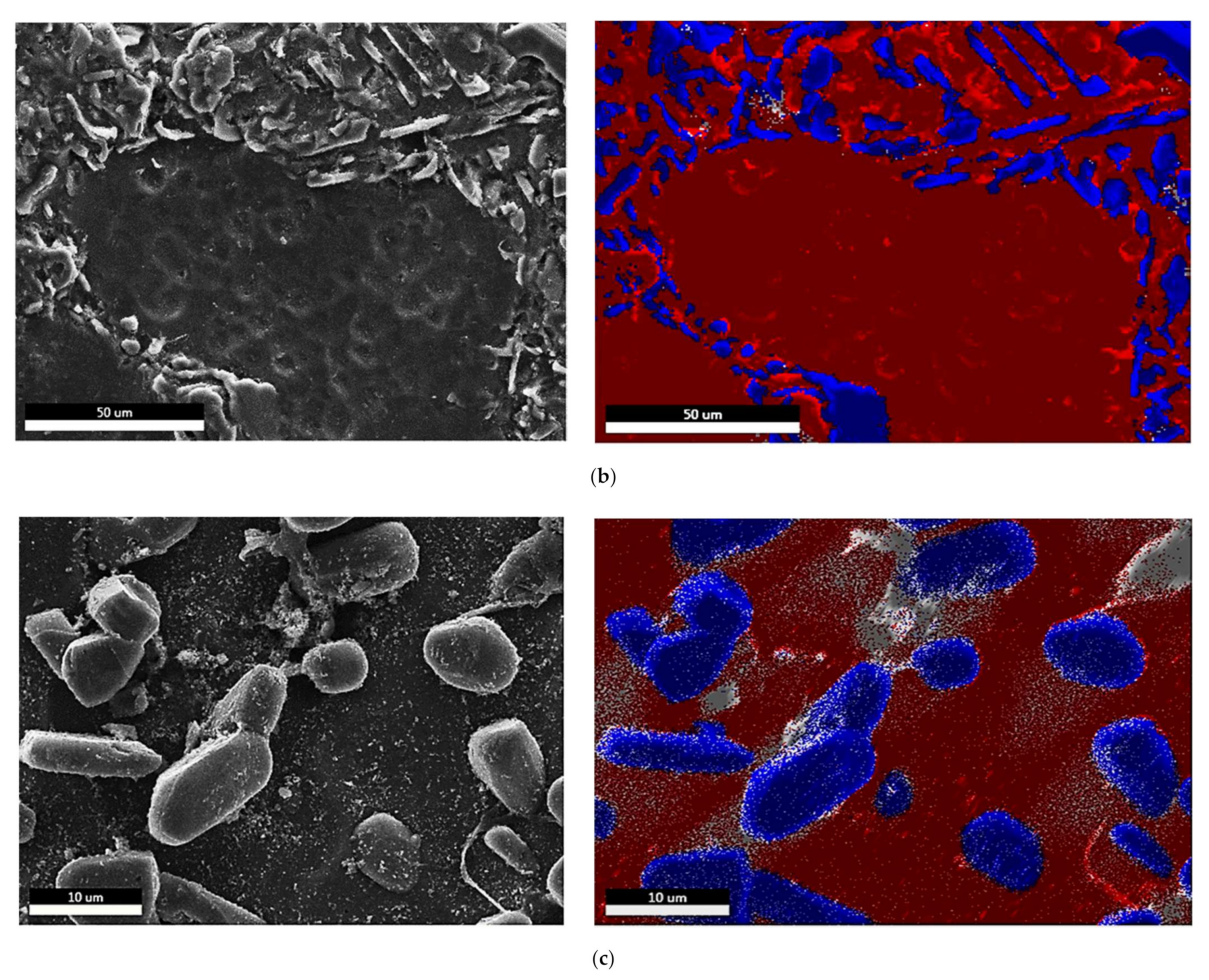
Electron images of deeply etched alloy samples showing element distribution (blue: Si particles, red background: Al matrix) in: (**a**) un-modified alloy—as cast, (**b**) Sr-modified alloy—as cast, (**c**) Sr-modified alloy—T6 tempered.

**Figure 12:**
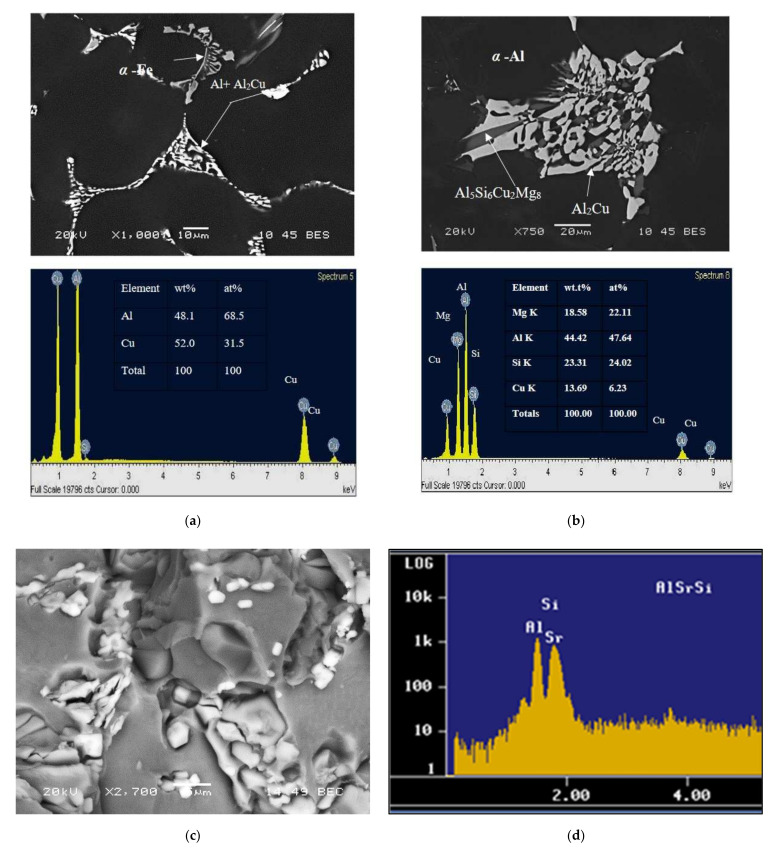
Backscattered electron micrographs and their corresponding EDX analyses, showing (**a**) segregation of the Al_2_Cu phase; (**b**) the formation of thick particles/platelets of Al_5_Si_6_Cu_2_Mg_8_ phase in grain-refined and heat-treated Al-11%Si alloys containing high levels of Cu and Sr, (**c**) precipitation of excesses of Sr, (**d**) EDS spectrum of Sr in black circle in (**c**).

Based on the statistical analysis, the corresponding hardness data indicated that the decrease in the hardness values of Sr-modified alloys compared to the non-modified alloys was mainly the result of changes in the morphology of the eutectic Si particles, from brittle coarse acicular plates in the non-modified alloy to a rounded fibrous form, as shown in Figure 10 and Figure 11. Furthermore, Sr led to a depression in the eutectic temperature, causing a shift of the eutectic point to a higher Si content, resulting in an increase in the amount of soft α-Al formed. It was also found that an increase in the Fe content resulted in a slight increase in the hardness values, which can be attributed to the formation of hard and brittle (metastable) intermetallic phases of Al_2_Cu and Al-Cu-Mg and also to an increased bonding of silicon particles with the matrix, in which the thermal energy is enough to precipitate such intermediate phases which are coherent with the matrix, as shown in the SEM micrograph and EDX analysis presented in Figure 12.

**Figure 13:**
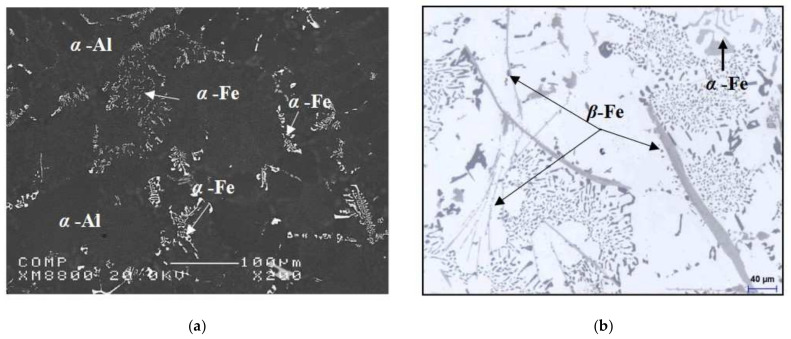
Backscattered and optical images showing the effects of the addition of Fe on the microstructure of grain-refined and heat-treated Al-11%Si alloys. (**a**) 0.5% Fe alloy (low Fe content); (**b**) 1.0% Fe alloy (high Fe content).

The results shown here prove that of the three alloying elements, Cu exerted the greatest effect by increasing the yield stress and the ultimate tensile strength values. We observed from the experiments that the elongation (%El) was highly sensitive to the alloy composition. With regard to the increased level of Cu in modified alloys, we found that the ductility was considerably lower. Such a result may be attributed to the influence of Sr on severity, as displayed by the Al_2_Cu phase segregation, resulting in the formation of large amounts of the coarse block-like form of the phase. It can also be noted that as the percentage of Fe increased beyond 0.75%, the elongation decreased to a significant degree, a fact which may be attributed to the presence of the β-Fe phase in the structure of the alloy, containing a high level of Fe, as shown clearly in Figure 13. The high stress concentrations at the sharp edges of the β-Fe phase, as well as the weak bonding between the β-phase and the Al matrix, enhance crack initiation and thus decrease the ductility of this alloy. This observation is in agreement with the work of Ojolo and Ogunkomaiy [23] who reported that increasing Cu and Mg contents generally increased strength and decreased ductility, whereas increasing the Fe content (at an Fe/Mn ratio = 0.5) dramatically lowered the ductility and strength of low-Si alloys.

The morphology of eutectic silicon in the Al–Si alloys has a major influence on the machining behavior. Through our analysis, we found that an increase in the Sr level has the greatest effect in terms of increasing the total cutting force and power values. In other words, the non-modified alloy (with a low level of Sr) generated lower drilling forces compared to Sr-modified alloys, which may be explained by the fact that the non-modified acicular silicon structure provided an easy path for fracture, resulting in decreases in the cutting forces during the machining. The higher drilling force and power observed with an increasing level of Cu and Fe may be attributed to an increase in the volume fraction of Cu and Fe intermetallics with the increase in the Cu and Fe content.

The interactions between the alloying elements play a prominent role in affecting the machining behavior of Al–Si casting alloys [24,25]. In the present study, the three interaction factors between the parameters had significant effects on tool life, including the interaction of the Cu content and Fe content (X_1_X_2_), the Cu content and Sr level (X_1_X_3_), and the Fe-content and Sr level (X_2_X_3_). This fact may be attributed to the formation of complex insoluble phases between Cu, Fe, Si, and Al, resulting in the formation of large amounts of coarse undissolved phases with an increase in Cu and Fe contents. From the machinability point of view, such undissolved phase particles represent the abrasive area of the matrix, with the potential to cause tool breakage. It has been reported that tool wear can be increased by as much as 50% through the presence of substantial quantities of undissolved Al–Cu and Al–Cu–Mg–Si phases. These results are in agreement with the work of Khorasani et al. [19], who reported that the dominant variables influencing tool life in the Al-Si alloys are the morphology of eutectic silicon; the inhomogeneities of the alloy structure; and an interrupted regime of cutting, resulting from the coarse undissolved particles.

**Figure 14:**
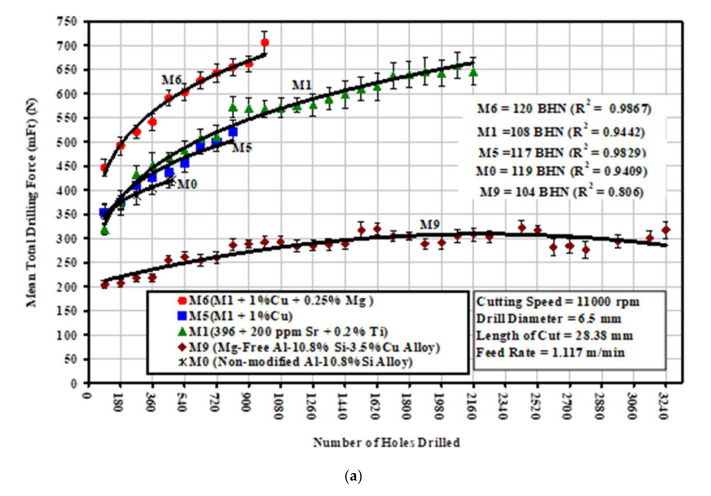

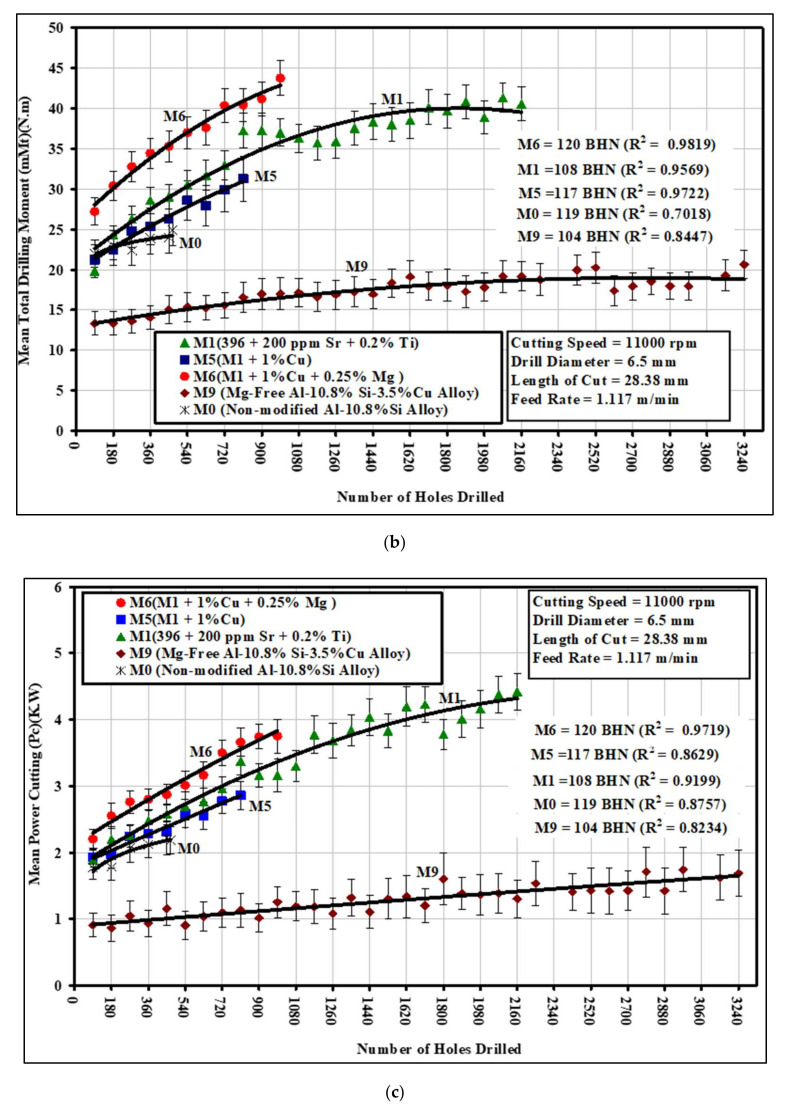
Effects of the addition of Cu, Mg, and Sr on the machinability of M1, M5, M6, M0, and M9 alloys in terms of (**a**) mean total drilling force, (**b**) mean total drilling moment, and (**c**) mean power cutting required for the drilling of 90 holes.

The machining characteristics of the Al-11%Si alloy depend mainly on the shape, size, and distribution of α-Al dendrites, the eutectic Si morphology, and Al_2_Cu particles in the interdendritic region. Figure 14a–c show that the addition of 1% Cu to the base alloy (coded the M1 alloy), thereby producing the M5 (M1 + 1.0% Cu) alloy, had only a slightly diminishing effect on the drilling force and moment, compared to the case of the M1 alloy. On the other hand, the increase in the level of Cu and Mg from 2.2% and 0.3% in the M1 alloy to 3.4% and 0.6%, creating the M6 alloy, had a noticeable effect in terms of increasing the mean total drilling force and mean total drilling moment, by 25% and 20%, respectively, compared to the M1 alloy. It can also be clearly observed that the Mg-free M1 alloy (coded the M9 alloy) displayed a significant decrease in the total drilling force and in the total drilling moment compared to the M1 reference alloy; specifically, the M9 alloy required an average of 50% lower mean total drilling force, ranging from 35% to 65%, and exhibited an average of 52% lower total drilling moment, ranging from 35% to 69%, as shown in Figure 14.

## 6. Conclusions

The selection of an alloy with certain specific properties is extremely exhausting and time consuming, particularly because the classic methods have not always led to the development of a quantitative relationship between the mechanical and machinability of the alloy on the one hand, and their chemical composition and melt treatments on the other. Therefore, if two or more variables are mofidied, it can become difficult to quantify the effect that any interaction between different variables would have on the alloy’s mechanical and machining properties. By using an experimental design (DOE) with only eight runs and the resulting regression equations, valuable information on the relationships of three independent variables—namely, Cu, Fe, and Sr contents—with the mechanical and machining properties of the near-eutectic T6-treated Al-11%Si alloy was obtained. Through an analysis of the results obtained, the following conclusions may be drawn:

1. Based on the statistical analysis, the corresponding hardness data indicated that the decrease in the hardness value of Sr-modified alloys compared to the non-modified alloys was mainly the result of changes in the morphology of the eutectic Si particles, from brittle coarse acicular plates in the non-modified alloy to a rounded fibrous form;
2. The results proved that of the three alloying elements, Cu had the greatest effect in terms of increasing the yield stress and ultimate tensile strength values. This fact may be attributed to the formation of the hard and brittle (metastable) intermetallic phases Al_2_Cu and Al–Cu–Mg. It was also found that an increase in the Fe content resulted in a slight increase in hardness values;
3. The elongation percentage of alloys was effected by three elements, with Fe and Cu having the greatest effect and Sr having the least;
4. The morphology of eutectic silicon in the Al–Si alloys has a major influence on the machining behavior. Through our analysis, we found that an increase in the Sr level had the greatest effect in terms of increasing the total cutting force and power values;
5. The higher drilling force and power with an increased level of Cu and Fe may be attributed to an increase in the volume fraction of Cu- and Fe-intermetallics with an increase in the Cu and Fe content;
6. The presence of a number of binary interactions indicated the formation of various intermetallic compounds. Therefore, several interaction effects were present, and they may have had the most significant effect on the tool life. These interaction effects included those of the Cu content and Fe content (X_1_X_2_), the Cu content and Sr level (X_1_X_3_), and the Fe content and Sr level (X_2_X_3_);
7. The validity of the equation was checked and the results indicated that there was a close match between the properties obtained by performing random experiments and those calculated by means of the respective regression equations. The closeness of the match indicates that the equations were sufficiently accurate over the range of variables.

## Figures and Tables

**Table 1:** Experimental settings for independent variables.

No.	Parameters	Notation	Unit	Level			
				Original		Coded	
				Low	High	Low	High
1	% Copper (Cu)-content	X_1_	wt%	2.25	3.5	−1	1
2	% Iron (Fe)-content	X_2_	wt%	0.5	1	−1	1
3	Strontium (Sr)-level	X_3_	ppm	0	200	−1	1

**Table 2:** Response variables and codes.

Response Variable	Unit	Code
Hardness	BHN	Y_1_
Yield stress (YS)	MPa	Y_2_
Ultimate tensile strength (UTS)	MPa	Y_3_
Elongation (El)	%	Y_4_
Total cutting force (Ft_m_)	N	Y_5_
Drilling power (Pc)	K.W	Y_6_
Tool life	No. of holes	Y_7_

**Table 3:** Cutting parameters applied for machinability testing.

Parameters	Drilling
Speed	234.5 m/min or 11,000 rpm
Drill type	Solid carbide “K20” drills: 6.5 mm
Hole depth	Length of cut ≤ 4.5 × D (28.38 mm)
Feed rate	1.117 m/min

**Table 4:** Experimental parameters and average response variables for trial experiments (runs) used for the factorial design.

	Independent Variable						Response Variable						
Run	Coded Value			Original Value			Y1 (Hardness)	Y2 (Y.S)	Y3 (UTS)	Y4 (%El)	Y5 (F_tm_) ^c^	Y6 (P_c_) ^c^	Y7 (Tool Life)
	X_1_	X_2_	X_3_	%Cu	%Fe	Sr							
1	−1	−1	−1	2.25	0.5	NM ^a^	117 ± 4.55	346 ± 4.7	382 ± 2.96	0.66 ± 0.47	422 ± 7.87	2.3 ± 0.21	468
2	1	−1	−1	3.5	0.5	NM	113 ± 3.76	320.39 ± 3.34	380.61 ± 1.75	0.57 ± 0.14	420 ± 6.24	2 ± 0.14	800
3	−1	1	−1	2.25	1	NM	116 ± 3.45	330.11 ± 5.92	360.78 ± 2.36	0.54 ± 0.09	410 ± 17.9	1.91 ± 0.25	637
4	1	1	−1	3.5	1	NM	110 ± 3.95	320.4 ± 6.13	339.5 ± 9.85	0.51 ± 0.02	460 ± 14.5	2.06 ± 0.24	1867
5	−1	−1	1	2.25	0.5	M ^b^	108 ± 3.56	358.1 ± 1.55	394.04 ± 6.27	1.05 ± 0.12	490 ± 10.1	2.74 ± 0.21	2160
6	1	−1	1	3.5	0.5	M	120 ± 3.19	355.94 ± 7.45	369.99 ± 8.54	0.72 ± 0.14	500 ± 13.5	2.85 ± 0.16	835
7	−1	1	1	2.25	1	M	111 ± 4.39	326.84 ± 2.13	354.72 ± 4.86	0.81 ± 0.04	524 ± 17.1	3.1 ± 0.34	971
8	1	1	1	3.5	1	M	122 ± 4.45	392.7 ± 6.8	400.72 ± 6.8	0.63 ± 0.02	600 ± 17.9	3.01 ± 0.21	1011

^a^ NM: Non-modified (0 Sr); ^b^ M: modified alloy with 200 ppm Sr; ^c^ (F_tm_) and Pc: total cutting force and power average for the first 180 holes in order to neglect the effect of tool wear on cutting force values.

**Table 5:** Multiple regression coefficients.

Response Variables	Coded	R^2^	$R_{Adj}^{2}$
Hardness	Y1	0.90997	0.85944
Yield Stress (YS)	Y2	0.8068	0.6136
Ultimate tensile strength (UTS)	Y3	0.9419	0.8839
Elongation (El)	Y4	0.9479	0.895857
Total cutting force (Ft_m_)	Y5	0.9352	0.87044
Drilling power (Pc)	Y6	0.9152	0.830563
Tool life	Y7	0.898129	0.796258

**Table 6:** Mechanical and machining properties values calculated using Equations (6)–(12).

Variables			Hardness BHN	YS MPa	UTS MPa	%El	Ftm (N)	Pc (Kw)	Tool Life
%Cu	%Fe	%Sr	Predicted Values						
2.35	0.70	0.02	118.838	405.92	439.136	0.5627	466	2.611	1527.67
2.5	0.9	0.02	119.016	418.91	451.739	0.5644	466.4	2.629	1699.19
2.45	0.5	0.02	119.072	398.65	432.493	0.5507	465.9	2.596	1404.47
3	0.6	0	119.7	414.2	449.2	0.531	463.5	2.603	1584.56

**Table 7:** Values of mechanical and machining properties obtained from random experiments.

Variables			Hardness BHN	YS MPa	UTS MPa	%El	Ftm (N)	Pc (Kw)	Tool Life
%Cu	%Fe	%Sr	Experimental Values						
2.35	0.70	0.02	115 ± 3.19	351.6 ± 2.75	390.5 ± 5.7	0.89 ± 0.15	488 ± 7.78	2.26 ± 0.2	1620
2.5	0.9	0.02	117 ± 3.95	320.4 ± 12.7	339 ± 9.85	0.51 ± 0.02	437 ± 6.24	2.08 ± 0.2	1867
2.45	0.5	0.02	110 ± 1.3	335.75 ± 5.76	371.7 ± 6.5	0.68 ± 0.13	401 ± 10.1	1.46 ± 0.2	1512
3.00	0.6	0	120 ± 3.45	346.11 ± 5.92	382.7 ± 2.36	0.84 ± 0.16	422 ± 13.5	2.3 ± 0.5	900

**Table 8:** Analysis of variance (ANOVA) for yield stress data (YS in MPa).

Source	Sum of Squares	DOF	Mean Square	Estimate Effects (%)	F-Ratio	*p*-Value	Remark
X1:%Cu	400.0	1	400.0	20	400.00	0.0000	Significant
X2:%Fe	110.25	1	110.25	10.5	110.25	0.0000	Significant
X3:Sr-level	240.25	1	240.25	15.5	240.25	0.0000	Significant
X_1_X_2_	1193.7	1	1193.7	34.55	1193.70	0.0000	Significant
X_1_X_3_	131.103	1	131.103	11.45	131.10	0.0000	Significant
X_2_X_3_	64.8025	1	64.8025	−8.05	64.80	0.0000	Significant
Total error	1.33554 × 10^−10^	0					
External sigma			1.0				
Total (corr.)	3671.69	6					

**Table 9:** Analysis of variance (ANOVA) for cutting force data (Ftm in N).

Source	Sum of Squares	DOF	Mean Square	Estimate Effects	F-Ratio	*p*-Value	Remark
X1:%Cu	1369.0	1	1369.0	37.0	1369.00	0.0000	Significant
X2:%Fe	1936.0	1	1936.0	44.0	1936.00	0.0000	Significant
X3:Sr-level	9409.0	1	9409.0	97.0	9409.00	0.0000	Significant
X_1_X_2_	1089.0	1	1089.0	33.0	1089.00	0.0000	Significant
X_1_X_3_	36.0	1	36.0	6.0	36.00	0.0000	Significant
X_2_X_3_	529.0	1	529.0	23.0	529.00	0.0000	Significant
Total error	1.16529 × 10^−10^	0					
External sigma			1.0				
Total (corr.)	28,694.9	6					

**Table 10:** Analysis of variance (ANOVA) for tool life data (number of holes).

Source	Sum of Squares	DOF	Mean Square	Estimate Effects	F-Ratio	*p*-Value	Remark
X1:%Cu	34,596.0	1	34,596.0	186.0	34,596.00	0.0000	Significant
X2:%Fe	29,756.2	1	29,756.2	172.0	29,756.25	0.0000	Significant
X3:Sr-level	34,040.3	1	34,040.3	184.0	34,040.25	0.0000	Significant
X_1_X_2_	465,806.0	1	465,806.0	682.5	465,806.25	0.0000	Significant
X_1_X_3_	686,412.0	1	686,412.0	−828.5	686,412.25	0.0000	Significant
X_2_X_3_	461,041.0	1	461,041.0	−679.0	461,041.00	0.0000	Significant
Total error	5.82077 × 10^−10^	0					
External sigma			1.0				
Total (corr.)	1.82659 × 10^−6^	6					

## Data Availability

Data will be made available upon request.
